# Exploring the value Australian community leaders see in a system dynamics model calibrated with local data: social norms and childhood obesity

**DOI:** 10.1136/bmjopen-2024-087195

**Published:** 2025-02-19

**Authors:** Loes Crielaard, Andrew D Brown, Mary Nicolaou, Joshua Hayward, Karien Stronks, Steven Allender

**Affiliations:** 1 Department of Public and Occupational Health, Amsterdam University Medical Centres, Amsterdam, The Netherlands; 2 Faculty of Health, Global Centre for Preventive Health and Nutrition (GLOBE), Institute for Health Transformation, Deakin University, Geelong, Victoria, Australia

**Keywords:** PUBLIC HEALTH, Obesity, Community-Based Participatory Research

## Abstract

**Abstract:**

**Objectives:**

Systems approaches (SAs) seek to understand the dynamics behind system behaviour and formulate effective actions given these dynamics. In public health, SAs often rely on qualitative systems maps visualising factors and their interconnections, frequently developed through group model building. Quantitative system dynamics models (SDMs) can offer additional insights: SDMs can simulate how system behaviour would change if we were to make an adjustment to the system, in what-if scenarios. We explored what (added) value Australian community leaders involved in SAs see in an SDM for understanding a system and its behaviour.

**Setting:**

The Whole of Systems Trial of Prevention Strategies for Childhood Obesity (WHOSTOPS), a community-level collaboration between researchers and community leaders in South-Western Victoria, Australia.

**Design:**

We calibrated an existing small and high-level SDM with local data from the WHOSTOPS communities, so that the simulations pertained to their local context. The SDM was developed to simulate potential interventions addressing either social norms regarding body weight or individual weight-related behaviour. We presented the SDM to the community leaders via an interactive interface in an online workshop.

**Participants:**

We calibrated the SDM using WHOSTOPS’ baseline measurement (2015), with an 80% participation rate among eligible children (1792/2516). 11 community leaders participated in the workshop.

**Results:**

The community leaders’ first impression of the SDM was that it could be a valuable additional tool, particularly because of its ability to compare what-if scenarios resembling individual vs systems perspectives, intuitive presentation of simulation results, and use of local data.

**Conclusions:**

Our preliminary exploration showed that the small and high-level SDM, using what-if scenarios reflecting interventions on different system levels, could contribute to the understanding and communication of (community-based) SAs.

**STRENGTHS AND LIMITATIONS OF THIS STUDY**

- This study unites three novel scientific contributions in the realm of systems approaches to obesity—the Whole of Systems Trial of Prevention Strategies for Childhood Obesity (WHOSTOPS) communities-researchers partnership, the WHOSTOPS data, and a system dynamics model studying social norms and obesity—and uses them as building blocks, where the incorporation of quantitative system dynamics modelling is still uncommon in community-based participatory system dynamics in public health.
- The WHOSTOPS communities-researchers partnership has been ongoing since 2015, meaning that the WHOSTOPS community leaders have become experts in and champions of systems approaches to childhood obesity, making this an excellent context to explore what (added) value stakeholders involved in systems approaches see in a system dynamics model for understanding a system and its behaviour.
- To make simulation feasible, it was necessary to merge some of the communities based on local government area (ie, geographically) for purposes of sample size—resulting in six communities as opposed to the 10 communities considered in other studies relating to WHOSTOPS.
- Because of their existing knowledge regarding systems approaches and their prior interest in system dynamics modelling, the WHOSTOPS community leaders’ first impression of the system dynamics model as a valuable additional tool should be corroborated by audiences unfamiliar with systems approaches and system dynamics modelling.

## Introduction

 The global increase in people living with obesity reflects the growth of a complex public health problem^1^ characterised by drivers acting at multiple levels ranging from individual metabolism to international policy and financial structures.^2^,^6^ While individual-level factors are well-understood, research has only recently started to consider how weight-related behaviour is heavily influenced by environment-level factors including food systems, market forces, and social norms.^6^,^8^ Interconnections between these factors create feedback effects, where the system may counteract individual-level action if it is unsupportive of healthy behaviour, while the behaviour of individuals may also undermine environment-level changes. For example, marketing of unhealthy food affects preferences for unhealthy food, influencing purchasing behaviour, which in turn feeds back to increase marketing budgets for unhealthy food.^6 9 10^ The notion that environment-level factors that interact with individual-level factors must be addressed to improve population health is central to systems approaches (SAs) in public health.^11^

Recent obesity prevention trials have begun experimenting with SAs,^412^,^16^ which seek to understand the dynamics behind system behaviour and formulate effective actions given these dynamics.^11 17 18^ One example is the Whole of Systems Trial of Prevention Strategies for Childhood Obesity (WHOSTOPS) in South-Western Victoria, Australia.^4 13^ WHOSTOPS is a partnership between researchers and leaders from collaborating communities to understand and design responses to the drivers of childhood obesity across multiple levels of society. Central to the WHOSTOPS intervention were group model building (GMB) workshops,^19^,^23^ which are frequently used as the basis of SAs.^3 14 17^ In these workshops, community leaders were asked to collectively develop qualitative systems maps, visualising how factors relevant to childhood obesity are interconnected and involved in feedback effects in their community.^3 4^

GMB, which prioritises involvement of stakeholders in SAs and emphasises group facilitation, is a growing branch of the system dynamics field and has proven powerful in facilitating learning about the problem and achieving commitment, consensus, and cohesion among a group of stakeholders.^21 24^ The method enables combining the factors that different stakeholders regard as relevant to the problem into one model.^25^ The resulting model becomes the product of the entire group, often specific to the frame of reference of that group, that is, unique to the situation in the local context of their organisation or community.^17^ While GMB can be employed to generate both quantitative and qualitative models, SAs in public health have mostly engaged with GMB as a method to develop qualitative systems maps.^12^ Specifically in relation to childhood obesity, it has recently been shown that as a result of GMB stakeholders gained new insights regarding the importance of “culture change, food supply chain, and a systems approach”.^19^ GMB is often praised for its ability to convey what it means to take a systems perspective to stakeholders, as the method guides them in developing their own thought processes on feedback effects between drivers of population health across multiple levels.^26^

System dynamics models (SDMs) are quantifications of systems maps, using equations to formalise the feedback effects between a system’s factors.^27^ Through these equations, the long-term effects of all this interconnectedness can be calculated and condensed into one outcome of interest,^11 28^ for example, prevalence of childhood obesity over time. SDMs can offer additional insights, complementary to those gained through systems maps and GMB, because they can simulate how system behaviour would change if we were to make an adjustment to the system, in what-if scenarios.^29^ These what-if scenarios can for example reflect the implementation of contrasting interventions, leading to quantitative estimations of what would happen to system behaviour if we did things differently.^28^ What makes SDMs distinctive is that they offer the opportunity to not only visualise “the structure of our systems” but also simulate “how (…) we [could] change the structure of systems to produce more of what we want and less of that which is undesirable”.^28^ SDMs have been referred to as “management flight simulators”, enabling users to virtually experiment with the consequences that their decisions might have for system behaviour.^27^

While systems maps and GMB can accomplish a range of purposes, it can be difficult to imagine how potential interventions—especially those on higher system levels such as the (social) environment level—could affect system behaviour based on a map of factors and interconnections.^30^ System dynamics modelling can concretise, through simulation, what the effects of intervening on higher system levels could be. It might therefore contribute to deepening stakeholders’ understanding of the behaviour of complex systems and the possible impacts that interventions on different system levels may have.

We explored what (added) value community leaders partnering on WHOSTOPS, considering their involvement in SAs, see in an SDM calibrated with local data for understanding a system and its behaviour. The community leaders’ prior experience with systems maps and GMB, through their communities’ participation in WHOSTOPS, means that they are able to critically reflect on the added value of this approach in understanding and communicating SAs to a larger audience. First, we calibrated an existing small and high-level SDM^31^ with local data from the WHOSTOPS communities, so that the simulations pertained to their local context (Step 1). This locally adapted SDM shows how the modelled mechanisms play out in a specific population (assuming that the mechanisms in the existing SDM are applicable to that local context). The existing SDM was developed to simulate potential interventions addressing either social norms regarding body weight or individual weight-related behaviour for an Amsterdam-based adult cohort,^31^ registered in the HELIUS study.^32^ Second, we organised an online workshop to present the SDM to a group of WHOSTOPS community leaders via an interactive interface, developed for this purpose (Step 2).

## Methods

### Step 1: Calibrating the system dynamics model with the WHOSTOPS data

#### Step 1A: The system dynamics model

The existing small and high-level SDM, previously published elsewhere,^31^ was developed to simulate how group-level median body mass index (BMI) would change in what-if scenarios corresponding to implementing an individual-level intervention, targeting weight-related behaviour, and implementing the same individual-level intervention combined with an environment-level intervention, targeting the social norm regarding body weight. The WHOSTOPS communities were recognised as “distinct, dispersed population centres agreed by partners based on existing government, health service, and education boundaries”,^4^ suggesting they can be defined as separate social groups with their own social norms.

**Figure 1:**
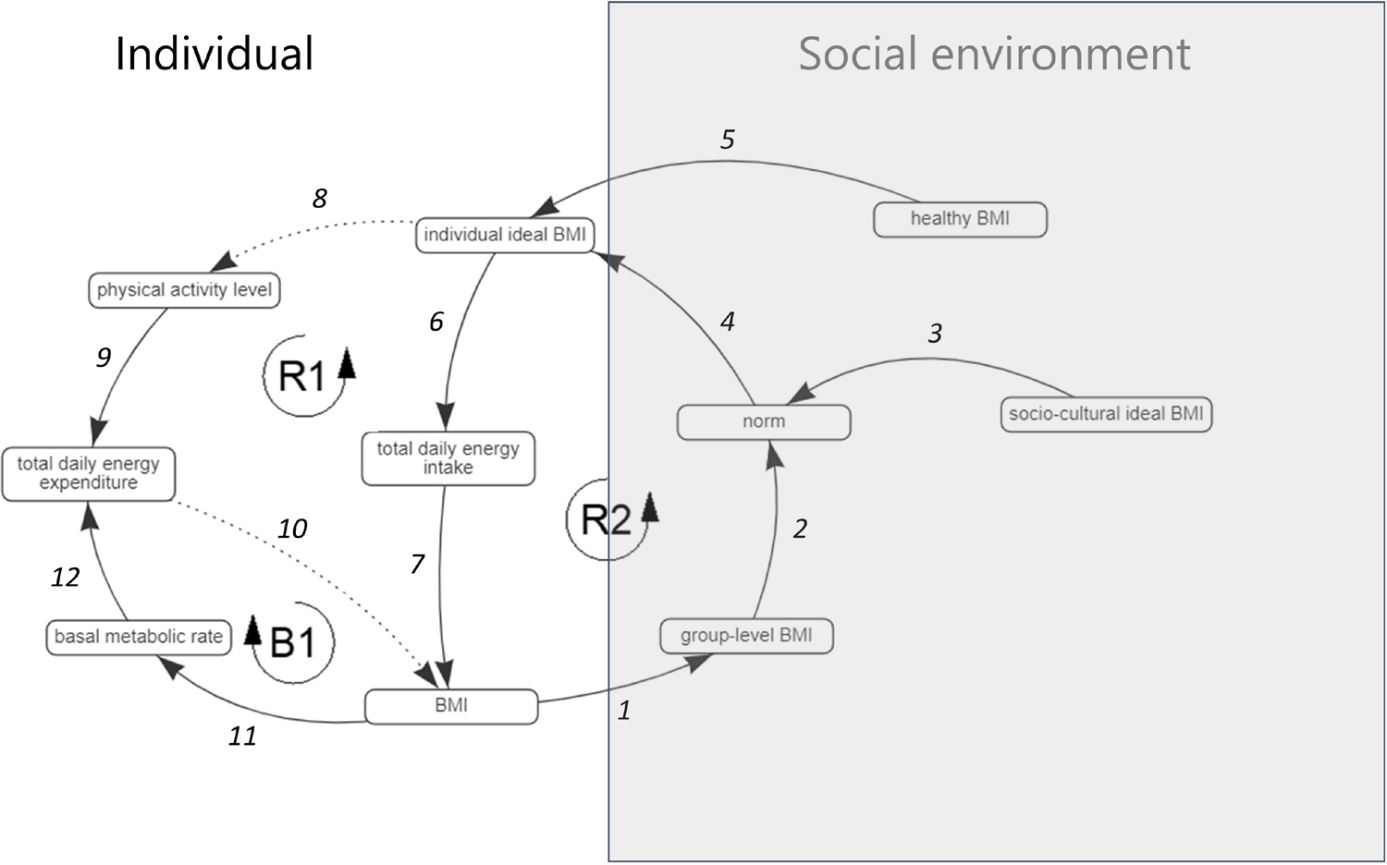
The qualitative systems map visualising the feedback effects captured in the system dynamics model. Causal links are numbered 1–12. Solid lines represent positive causal effects (an increase in the first factor causes an increase in the second factor) and dashed lines represent negative causal effects (an increase in the first factor causes a decrease in the second factor).

To estimate change in group-level median BMI in different what-if scenarios, the SDM simulates feedback effects between a group-level social norm—that is, the BMI that is regarded as normal—and individual eating and physical activity behaviour (figure 1).^31^ The social norm is determined equally by two components: median BMI measured at the group-level (link 2) and an individual’s own sociocultural ideal BMI (link 3).^31^ Group-level median BMI represents the BMI that is most prevalent in the group, while sociocultural ideal BMI represents “the relatively stable sociocultural perception an individual has of the ideal BMI in their group, which induces an individual variation in how the norm is regarded”.^31^ We thus take the social norm, that an individual adheres to, to be determined partly by what they see around them, which can change quickly, and partly by what they have internalised as being desirable, understood as a more ingrained personal belief based on cultural values.

The SDM represents that if the BMI regarded as normal increases, individual eating and physical activity behaviour will adjust to meet the social norm—that is, energy intake increases (reinforcing feedback loop 2 (R2), links 4-6-7-1-2) and energy expenditure decreases (R1, links 4-8-9-10-1-2)—while in turn increasing the social norm BMI. The social norm may thus make it easy or difficult for individuals to adopt healthy behaviour, while individual behaviour also contributes to the social norm. Balancing feedback loop 1 (B1) (links 11-12-10) indicates that more energy is required to sustain body function at rest when BMI is higher.^33^ Besides the social norm, the BMI regarded appropriate by each individual—that is, individual ideal BMI—may be influenced by knowledge about what a healthy BMI is (‘healthy BMI’, link 5). By activating knowledge of what constitutes a healthy BMI as an influence on individual ideal BMI, the SDM emulates the individual-level intervention.

Table 1 outlines the SDM’s equations. A full description is published elsewhere.^31^ Note that equation (8) was adjusted, compared with the existing SDM, to be applicable to children^33^ (p37, Table 5.2). Values imported into the SDM for each individual included gender, age, weight, and height, which were directly extracted from the WHOSTOPS data, and physical activity level, healthy BMI, total daily energy intake, and sociocultural ideal BMI, which were estimated from the WHOSTOPS data (procedures in online supplemental material). The SDM (implemented in Python) simulates group-level median BMI over 36 months of intervention with 95% confidence intervals (CIs).^31^ Note that the time scale (ie, the duration of the change in group-level median BMI) is not exact: what is of interest is the stable behaviour with respect to group-level median BMI that the system attains in the different what-if scenarios.^31^

**Table 1:** The system dynamics model’s equations

Equation					Causal links
(**1**)	${IIB}_{t}=0.5{\times HB+0.5\times Norm}_{t}$				4; 5
(**2**)	${{Discrepancy}_{BMIandIIB}}_{t}={BMI}_{t}-{IIB}_{t}$				
(**3**)	${PAL}_{t+1}={PAL}_{t=0}+{Intent}_{PAB}\times {{Discrepancy}_{BMIandIIB}}_{t}$				8
(**4**)	${TDEI}_{t+1}={TDEI}_{t=0}+{Intent}_{EB}\times {{Discrepancy}_{BMIandIIB}}_{t}$				6
(**5**)	${TDEE}_{t+1}={BMR}_{t}\times {PAL}_{t+1}$				9; 12
(**6**)	$\begin{array}{l} W_{t+1}=W_{t}+\left(Rate_{Weight\;gain}-Rate_{Weight\;loss}\right)\\ \;=W_{t}+\left(\frac{TMEI_{t+1}}{7700}-\frac{TMEE_{t+1}}{7700}\right)\\ \;=W_{t}+\;\left(\frac{TDEI_{t+1}}{7700}\times \frac{365}{12}-\frac{TDEE_{t+1}}{7700}\times \frac{365}{12}\right)\\ \;=W_{t}+\left(\frac{73\times TDEI_{t+1}}{18480}-\frac{73\times TDEE_{t+1}}{18480}\right) \end{array}$				7; 10
(**7**)	$BMI_{t+1}=\frac{W_{t+1}}{H_{t=0}^{2}}$				
(**8**)	${BMR}_{t+1}$	Boys	${Age}_{t=0}$3–9	${BMR}_{t+1}=22.706\times W_{t+1}+504.3$	11
			${Age}_{t=0}$10–18	${BMR}_{t+1}=17.686\times W_{t+1}+658.2$	
		Girls	${Age}_{t=0}$3–9	${BMR}_{t+1}=20.315\times W_{t+1}+485.9$	
			${Age}_{t=0}$10–18	${BMR}_{t+1}=13.384\times W_{t+1}+692.6$	
(**9**)	${MedianBMI}_{t+1}=Median({BMI}_{t+1})$				1
(**10**)	${Norm}_{t+1}=\frac{{MedianBMI}_{t+1}+{SCIB}_{t=0}}{2}$				2; 3

BMIbody mass indexBMRbasal metabolic rateDiscrepancy_BMIandIIB_discrepancy between BMI and individual ideal BMIHheightHBhealthy BMIIIBindividual ideal BMIIntent_EB_intent to change eating behaviourIntent_PAB_intent to change physical activity behaviourNormgroup-level social normPALphysical activity levelRate_Weight gain_weight gain rateRate_Weight loss_weight loss rateSCIBsociocultural ideal BMITDEEtotal daily energy expenditureTDEItotal daily energy intakeTMEEtotal monthly energy expenditureTMEItotal monthly energy intakeWweight

#### Step 1B: The WHOSTOPS data

WHOSTOPS involved collecting data in collaborating communities employing an opt-out approach.^4 13^ We used the baseline measurement (2015), with an 80% participation rate among eligible children (1792/2516 invited), to calibrate the SDM. A full description of WHOSTOPS can be found in the protocol^4^ and results^13^ papers.

Measurements (details in online supplemental material) used to calibrate the SDM included gender, age, weight, height, physical activity, and weight perception and concerned children in grades four (mean age 10 years) and six (12 years). Children with missing values for weight (0.6%), height (0.5%), physical activity (0.4%), and weight perception (4.3%) were excluded, leaving 1085/1141 children (4.9% excluded) (table 2).

**Table 2:** The baseline descriptives for the Whole of Systems Trial of Prevention Strategies for Childhood Obesity (WHOSTOPS) communities

		Community 1	Community 2	Community 3	Community 4	Community 5	Community 6	Pooled
		n=155	n=208	n=43	n=156	n=201	n=322	n=1085
Age (years), median (IQR)		10 (9–11)	11 (9–12)	11 (9.5–11)	10 (9–11)	10 (9–11)	10 (9–11)	**10 (9–11**)
BMI (kg/m^2^), median (IQR)		18.8 (16.7–21.8)	18.5 (16.7–21.5)	18.1 (16.2–21)	18.4 (17.2–21.4)	18.1 (16.4–21.1)	18.2 (16.5–20.5)	**18.4 (16.6–21.2**)
BMI≥19.2 kg/m^2^ (%)		46.5	44.2	37.2	41.0	39.8	38.5	**41.3**
Sociocultural ideal BMI (kg/m^2^), median (IQR)		18.7 (16.9–20.9)	18.0 (16.6–20.5)	18.3 (16.2–20.3)	18.3 (17.2–20.1)	18.1 (16.6–20.1)	18.1 (16.5–19.9)	**18.1 (16.7–20.2**)
Group-level social norm (kg/m^2^), median (IQR)		18.7 (17.8–19.8)	18.2 (17.6–19.5)	18.2(17.2–19.2)	18.4 (17.8–19.3)	18.1 (17.4–19.1)	18.1 (17.4–19.1)	**18.3 (17.5–19.3**)
Discrepancy between BMI and sociocultural ideal BMI	<0 (%)	14.2	14.9	7.0	14.7	18.4	14.3	**14.9**
	=0 (%)	61.9	56.7	81.4	60.9	55.2	65.2	**61.3**
	>0 (%)	23.9	28.4	11.6	24.4	26.4	20.5	**23.8**
Physical activity level	1.46 (%)	69.0	63.5	76.7	67.9	69.7	59.6	**65.4**
	1.7 (%)	31.0	36.5	23.3	32.1	30.3	40.4	**34.6**
Total daily energy intake (kcal), median (IQR)		2033.7 (1840.9–2273.0)	2055.2 (1870.7–2332.7)	1955.5 (1756.7–2123.2)	2002.1 (1798.5–2301.9)	1956.0 (1780.8–2204.3)	2031.1 (1804.8–2248.4)	**2010.6(1814.2–2274.0)**
Basal metabolic rate (kcal), median (IQR)		1328.9 (1213.8–1455.9)	1330.3 (1199.8–1466.1)	1275.8 (1192.2–1440.8)	1307.2 (1200.7–1466.2)	1297.9 (1186.1–1412.5)	1280.2 (1187.7–1410.6)	**1305.6(1192.9–1434.1)**

Note: Medians for all baseline descriptives are reported so as to be comparable to the outcome of interest group-level median BMI. The cut-off point for overweight (19.2 kg/m2) was calculated as a weighted average based on distribution of gender and age across the sample using BMI-for-age growth reference tables by the World Health OrganizationWorld Health Organization^43^ (values taken from the 1stfirst month of an age group) and was identical for all children. If Ddiscrepancy between BMI and socio-cultural ideal BMI<0, BMI is smaller than socio-cultural ideal BMI; if Ddiscrepancy between BMI and socio-cultural ideal BMI=0, BMI is equal to socio-cultural ideal BMI; if Ddiscrepancy between BMI and socio-cultural ideal BMI>0, BMI is larger than socio-cultural ideal BMI.

The numbers in bold show the baseline descriptives for the pooled data across the communities.

BMIbody mass index

By calibrating the SDM with data from the WHOSTOPS communities, we assumed that the mechanisms at its basis are also in effect in these communities. Because only the basal metabolic rate equations (to be applicable to children), the input data, and the population under study were modified, the locally adapted SDM is subject to the same assumptions as the existing SDM that were described in detail elsewhere.^31^

**Figure 2:**
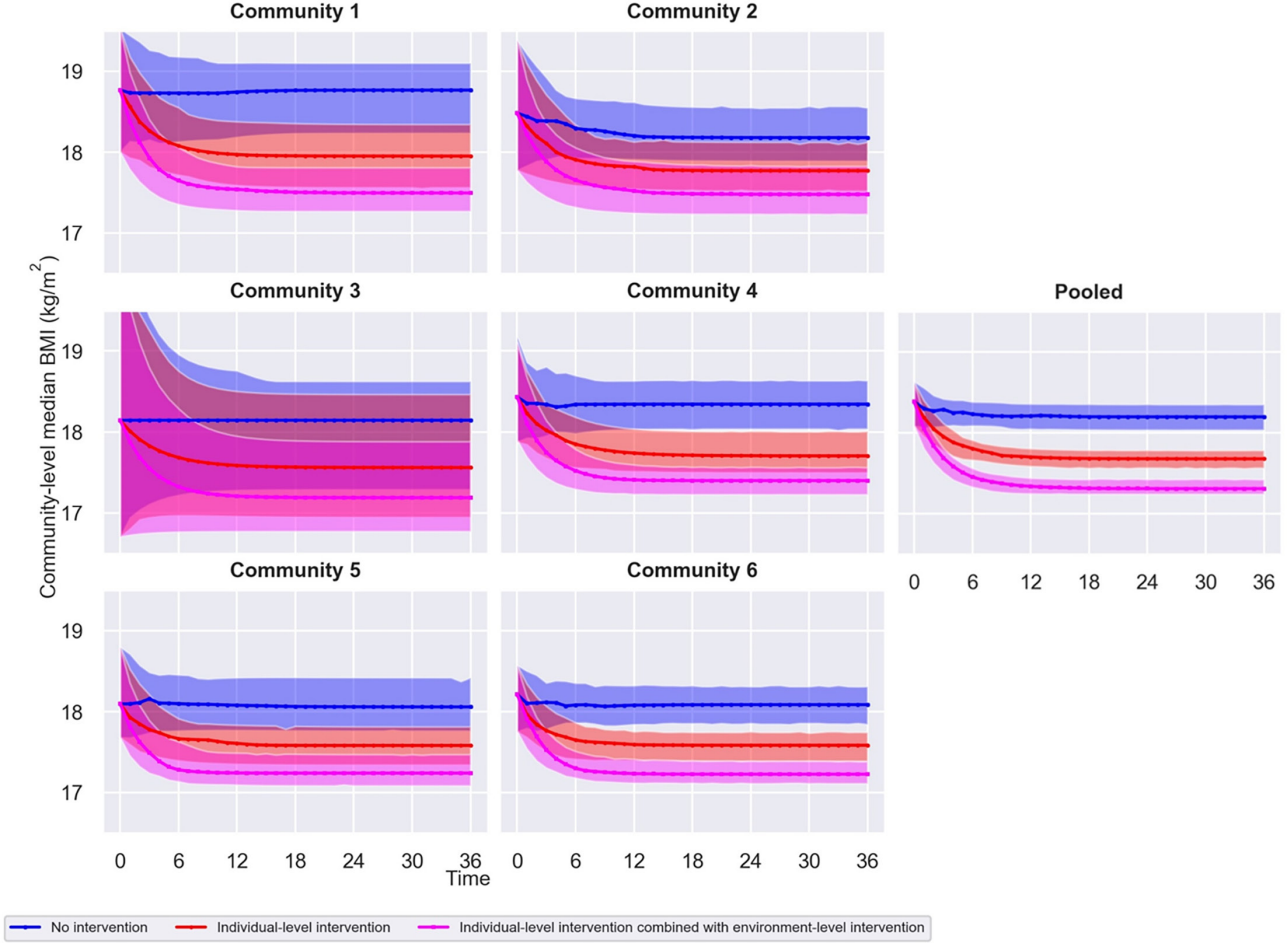
The simulation results. Estimated change in group-level median body mass index (BMI) for the Whole of Systems Trial of Prevention Strategies for Childhood Obesity (WHOSTOPS) communities, with 95% confidence intervals (CIs), in the what-if scenarios corresponding to no intervention (individual ideal BMI 100% determined by the (in this case unhealthy) social norm), the individual-level intervention (individual ideal BMI 50% determined by the social norm and 50% determined by knowledge of healthy BMI), and the same individual-level intervention combined with the environment-level intervention (individual ideal BMI 100% determined by knowledge of healthy BMI). Given the consistent pattern across communities, data were also pooled for simulation, leading to a more evident pattern and smaller CIs. Note that, compared with the existing system dynamics model, in the individual-level intervention what-if scenario, individual ideal BMI is preset to be for 50% determined by healthy BMI and for 50% determined by the social norm (see equation (1) in table 1).

Figure 2 shows the estimated change in group-level median BMI for the WHOSTOPS communities, with 95% CIs, in the what-if scenarios corresponding to no intervention, the individual-level intervention, and the same individual-level intervention combined with the environment-level intervention. Simulation results indicated that unhealthy social norms in the WHOSTOPS communities could diminish the effectiveness of individual-level interventions targeting weight-related behaviour by approximately 35% on average. This implies that in the WHOSTOPS communities, similarly as in Amsterdam, the social environment—in the form of the social norm—counteracts individual-level action.

### Step 2: Presenting the system dynamics model to community leaders partnering on WHOSTOPS

#### Step 2A: The interface

**Figure 3:**
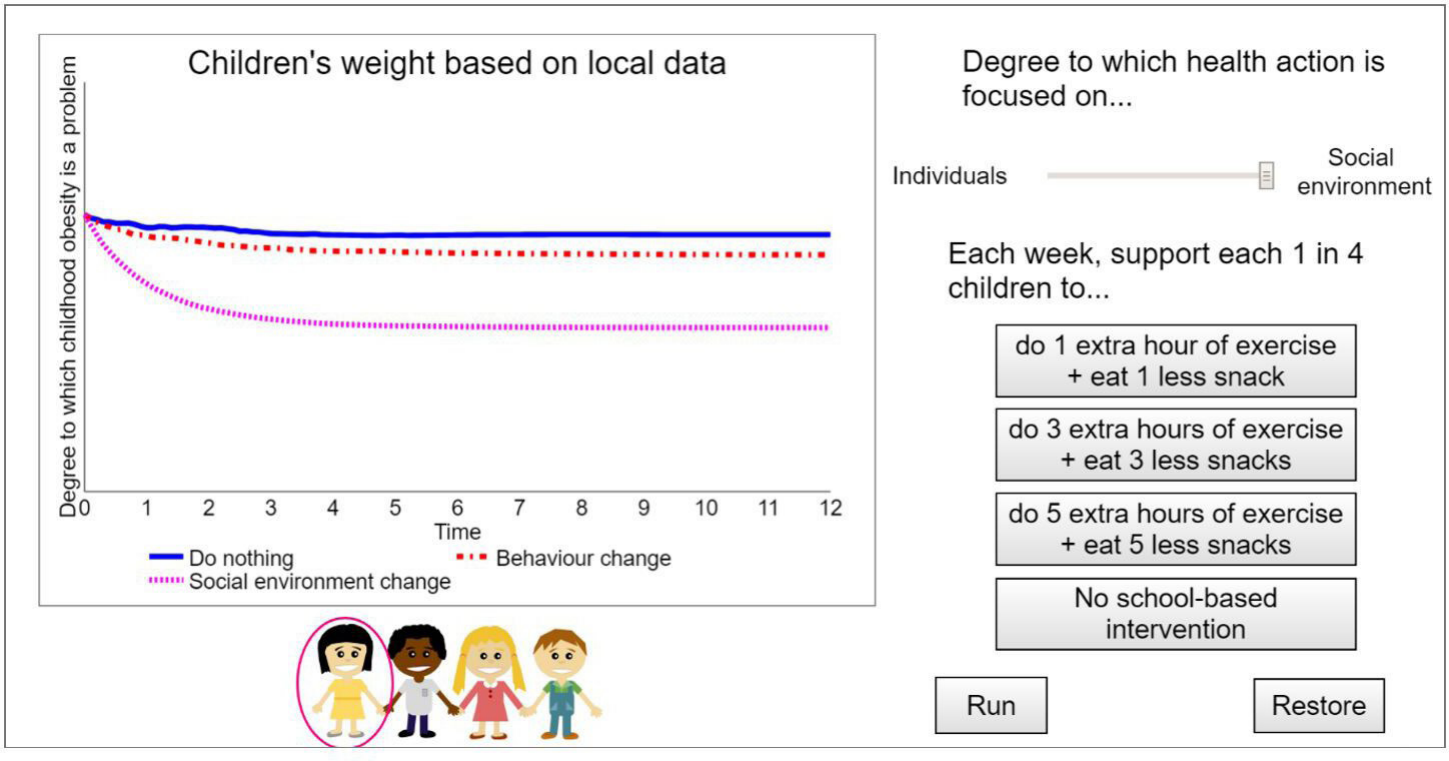
The Stella interface. The simulation results of setting the slider to *Individuals* and clicking the *No school-based intervention* button are plotted as *Do nothing*. Those of setting the slider to *Individuals* and clicking the *1 extra hour of exercise+1 less snack* button are plotted as *Behaviour change*, that is, only an individual-level intervention. Those of setting the slider to *Social environment* are plotted as *Social environment change*, that is, an individual-level intervention combined with an environment-level intervention. The *Run* and *Restore* buttons are used to start the simulation and clear the screen, respectively. The interface presents simulation results in real time (95% confidence intervals not included).

We prepared an interactive interface to allow users to engage with and test alternate settings of the SDM with Stella Version 2.0.3^34^ (figure 3). Using the slider, change in group-level median BMI (*Degree to which childhood obesity is a problem*) in the what-if scenarios of the existing SDM^31^ can be simulated. Setting the slider to *Individuals* resembles implementing no intervention while keeping the influence of the (in this case unhealthy) social norm constant. This corresponds to letting individual ideal BMI be for 100% (instead of 50%) determined by the social norm in equation (1) (table 1). In contrast, setting the slider to *Social environment* resembles implementing the individual-level intervention (activating knowledge of healthy BMI as an influence) combined with the environment-level intervention (deactivating the influence of the social norm). If the social norm is unhealthy and its influence is deactivated, this emulates creating a social environment supportive of healthy behaviour. This corresponds to letting individual ideal BMI be for 100% (instead of 50%) determined by knowledge of healthy BMI in equation (1). Setting the slider anywhere in between *Individuals* and *Social environment* means tweaking the ratio of the influence of the social norm vs knowledge of healthy BMI.

For this interface, the what-if scenarios of the existing SDM were extended to include interventions that directly target eating and physical activity behaviour, where interventions of this type were considered most familiar. Using the buttons, change in group-level median BMI in what-if scenarios corresponding to implementing one of three individual-level interventions, ranging from low to high intensity, or no intervention can be simulated. These individual-level interventions were assumed to be school-based and adhered to by one in four children in the community. Intervention effects on total daily energy intake and expenditure were estimated based on the Human Energy Requirements report: 1 extra hour of exercise=0.175 extra physical activity level units (incorporated in equation (3), table 1) and 1 less snack=200 less kilocalories (incorporated in equation (4)).^33^

To illustrate change in group-level median BMI in different what-if scenarios, we first presented simulation results of setting the slider to *Individuals* and clicking the *No school-based intervention* button to the community leaders—plotted as *Do nothing* (figure 3). We then showed them simulation results of keeping the slider on *Individuals*, while adding an individual-level intervention by clicking the *1 extra hour of exercise+1 less snack* button—plotted as *Behaviour change*. Lastly, we set the slider to *Social environment*, corresponding to activating knowledge of healthy BMI as an influence and deactivating the influence of the social norm—plotted as *Social environment change*.

#### Step 2B: The workshop participants and recruitment

The 2-hour workshop with the WHOSTOPS community leaders took place in May 2022 as a securely recorded and stored Zoom session. Approximately 100 community leaders were invited based on their past participation in SAs research and consent to being contacted for follow-up research. Community leaders are defined as such because of their professional role—in for example, a health service, community-based organisation, or local business—or involvement with community volunteering. The 11 participating community leaders had roles related to health in local councils, health services, and utility companies. Four of the six communities for which simulation results were generated were represented by the participating community leaders. Full ethics clearances for the workshop were received from: Deakin University’s Human Ethics Advisory Group-Health (HEAG-H) 7_2022. Patients or the public were not involved in the design, conduct, reporting, or dissemination plans of our research.

#### Step 2C: The workshop procedure

The workshop agenda was based on the Model Review, Concept Model, and Action Ideas GMB scripts available on Scriptapedia,^35^ involved one modeller (LC), one facilitator (ADB), and three group moderators (MN, JH, SA), and consisted of four sections. First, the qualitative systems map underlying the SDM^31^ (figure 1) was explained, emphasising feedback effects and system behaviour (Model Review). Second, the SDM’s equations and calibration with the WHOSTOPS data were described. Third, community leaders were shown a subset of the pooled simulation results via the interface by tracing system behaviour over time in different what-if scenarios (Concept Model) (figure 3). Fourth, community leaders were divided into groups (of three/four, accompanied by one/two researcher(s)) based on geographical location and encouraged to interact with the interface—by asking a researcher to select settings—and express their thoughts, after which all community leaders were invited to collectively discuss feedback on and potential practical applications of the interface (Action Ideas).

#### Step 2D: The workshop analysis

Zoom recordings covered the entire workshop, where comments made during all sections were taken into account. Central to the analysis was the fourth section (Action Ideas) described above. Recordings were transcribed verbatim for analysis. Synthesis of transcripts used the six-phase process for thematic analysis: “familiarising yourself with your data” (Phase 1), “generating initial codes” (2), “searching for themes” (3), “reviewing themes” (4), “defining and naming themes” (5), and “producing the report” (6).^36^ Themes were extracted by LC and verified by ADB, after which LC and KS condensed them into three key aspects that made the WHOSTOPS community leaders consider the SDM as a valuable additional tool, presented in detail below.

### Patient and public involvement

None.

## Results

The WHOSTOPS community leaders described the SDM’s emphasis on social norms as highly relevant for illustrating that environment-level factors must be addressed to improve population health.^11^ Prompted about social norms affecting health in their community, they gave examples about “what a healthy weight is”, “everyone wants their kids to be healthy”, and “eating healthy is almost uncool”. The community leaders’ impression of the SDM was that it could be a valuable additional tool, particularly because of its ability to compare what-if scenarios resembling individual vs systems perspectives, intuitive presentation of simulation results, and incorporation of local data.

### Ability to compare what-if scenarios resembling individual vs systems perspectives

The community leaders were positive about the SDM’s ability to compare interventions to change group-level median BMI based on different conceptual representations, emphasising individual-level vs environment-level factors, of how this complex public health problem works.

*I thought I had seen some system dynamics type modelling where things are kind of turned off and on (…)[which]changes the outcome, but (…) this has been completely new to me: comparing different solutions to change that same outcome.*

That these what-if scenarios resembled different perspectives was described as useful for third-party local stakeholders in grasping why actions at an environment-level scale, taking account of feedback effects, may have more impact. This was pointed out to be a demonstration of Meadows’ Places to Intervene in a System.^28 37^

*I am comparing it to something like (…) Donella Meadows’levels of systems change: (…) a snapshot of (…) at what level the actions actually have impact and (…) to shift that thinking it is probably a helpful tool.*

The SDM was viewed as having the potential to “shift people’s thinking” from “short-term project-based individual work” focused on individual behaviour, seen as “quick” and “easy”, to the social environment.

*I see it as a really great communication tool to (…) get people looking at the focus of their strategic planning (…) and starting to see whether that (…) short-term project-based individual work could shift (…) into something more around social environment and influencing that aspect and that[ie,thesystem dynamics model]could really shift people’s thinking.*

*I think it is often seen as working at that individual level is: it is quick, it is easy, so there is a tendency to go there, whereas[we are]trying to get that support for broader-level work and the understanding of why the work that we are doing is important and it is needed. This would present I guess like some more evidence for why we need to do that.*

The community leaders were aware that this SDM was developed to demonstrate how social norms and childhood obesity interact only and were convinced about its utility for that objective. They noted that if the SDM were to be used as a decision-making tool or to secure funding, the environment-level intervention should be described in more detail. In other words, they expressed that the SDM is effective in exemplifying *why* targeting environmental-level factors is required but that an additional step is needed to understand *what* such an SA should exactly entail. To use the SDM as a decision-making tool, it was advised to have what-if scenarios be selected by stakeholders themselves and to accommodate changes to the SDM, for example, incorporating additional factors, so as to meet their needs.

### Intuitive presentation of simulation results

*I think (…) it would be a really good tool for people to understand (…) the impacts of those individualvsthose social environment aspects, like it has got that visual (…). And so I think in terms of, to us as health promoters, it seems really obvious why you would focus on those social environment aspects, but often to other people, whether it is other people within health services, other stakeholders, right down to the community, they do not have that understanding, so this gives them that (…) idea, (…) it is a visual cue, (…) which I think is really positive.*

The community leaders described the presentation of simulation results as “a visual cue”, “relatable”, “convincing”, and “powerful”. Showing an interface with “just (…) one graph” was thought to be less overwhelming for stakeholders than presenting a large amount of data. The community leaders believed that this presentation invites discussion and “creates a clear goal for a holistic approach”. They noted that “seeing things (…) in a very simplistic manner” would help stakeholders without a background in SAs to understand the aim of and commitment around such approaches: the intuitive presentation could result in “aha moment[s]”. The interface being interactive was perceived as key in making it powerful.

*I think that(the)interactiveness[sic]of it, it is not just sitting there like a PowerPoint and saying you know this is why we are doing this as published in this journal, it is actually like a tool (…) that we can use in a presentation or in a meeting or in a consultation.*

Its simplicity and interactivity could also make the SDM relevant as an “experiential learning” tool, where stakeholders could be invited to interact with the interface. The community leaders emphasised that the SDM could serve to demonstrate the necessity of addressing environment-level factors particularly in settings where there is limited time to explain what it means to take a systems perspective, because it can illustrate the implications of focusing on individual behaviour or the social environment. They agreed that this presentation of simulation results should not become a replacement for community-based participatory system dynamics, qualitative systems maps, and particularly GMB, but can complement them or, in case of time constraints, serve as a tool that can exemplify *why* targeting environment-level factors is required.

*And we really made the distinction that it is not agroup model buildingsession which allows that build of understanding and transformation of thinking from individual to system (…). It is not replacing agroup model buildingsession, it is actually potentially if you're in a short meeting with a group of managers. You know, you could talk through (…) the advantages of and the distinction between doing individual focus programsvssystems change programs and it is a really strong advocacy piece. That we saw some real value in.*

It was mentioned that for the presentation of simulation results to be intuitive, the wording should be tailored to the setting at hand, where referring to BMI may be overly clinical or present a risk of stigma.

### Incorporation of local data

The community leaders recognised simulation as a way to use local data to move conversations beyond the focus on descriptives to looking ahead, something they are not accustomed to. They also agreed that the SDM being “evidence-driven” contributed to its usefulness in communicating the potential of SAs, by giving their communities “an idea of where we could end up”. The SDM being calibrated with local data was also described as helpful when applying for funding, speaking to stakeholders that want to see numbers by concretising “where you’re hopefully seeing yourself or your community in the future”. The SDM’s focus on social norms was seen as instrumental in promoting ownership of the problem among stakeholders outside of health.

*It could be a really valuable tool in terms of getting other stakeholders outside of health to understand why their input is important and why they need to be involved.*

Moreover, the SDM could build on the sense of responsibility achieved by community-based participatory system dynamics.

*I think it helps add to the[qualitative]systems map (…): everyone sees a system and goes, oh yeah, there are so many points for leverage and (…) it would be great if we got together and did something. But I think this might actually show the value of everyone actually following through and not just (…) saying that it would be good to do that.*

## Discussion

### Main findings

We explored what (added) value Australian community leaders involved in SAs see in an SDM calibrated with local data for understanding a system and its behaviour. Their first impression was that the SDM could be a valuable additional tool, particularly because of its ability to compare what-if scenarios resembling individual vs systems perspectives, intuitive presentation of simulation results, and incorporation of local data. The SDM’s focus on social norms was viewed as a meaningful example of the interaction between the social environment and individual behaviour and the need for addressing both in interventions. The what-if scenarios associated with the SDM were considered illustrative of what could actually happen if we intervened on higher system levels, supporting understanding and communicating the potential of (community-based) SAs.

### Strengths and limitations

This study unites three novel scientific contributions in the realm of SAs to obesity—the WHOSTOPS communities-researchers partnership, the WHOSTOPS data, and the SDM studying social norms and obesity—and uses them as building blocks, where the incorporation of quantitative system dynamics modelling is still uncommon in community-based participatory system dynamics in public health.^23^ Our findings should be considered in light of a number of strengths and limitations. First, the partnership between community leaders and researchers that is WHOSTOPS has been ongoing since 2015, meaning that the WHOSTOPS community leaders have become experts in and champions of SAs to childhood obesity. This provided an excellent context to explore what (added) value stakeholders involved in SAs see in an SDM. Still, because of their existing knowledge, the WHOSTOPS community leaders’ first impression of the SDM as a valuable additional tool should be corroborated by audiences unfamiliar with SAs. Analogously, the WHOSTOPS community leaders that attended the workshop were presumably those that were most interested in system dynamics modelling: 11 of a potential 100 participants chose to attend. The sample is thus neither representative for a general audience, nor for all WHOSTOPS community leaders. Second, WHOSTOPS combined community-based participatory system dynamics with data collection: this enabled the presentation of simulation results pertaining to the local context, while the opt-out approach made simulation results as representative as possible of the communities. An important limitation of the simulation results for the Amsterdam-based adult cohort (in which context the SDM applied here was developed) was that the social groups sharing a social norm needed to be decided on a priori based on shared characteristics—because information about the communities that individuals were embedded in was lacking.^31^ In contrast, WHOSTOPS followed individuals within their own communities, meaning that the simulation results are more likely to be reflective of the actual social environment. A limitation is that, to make simulation feasible, it was necessary to merge some of the communities based on local government area (ie, geographically) for purposes of sample size—resulting in six communities as opposed to the 10 communities considered in other studies relating to WHOSTOPS. Still, this grouping, implying that some communities share a social norm regarding body weight, was made in agreement with WHOSTOPS researchers.

### Implications for policy and practice

The application of SAs and in particular the use of quantitative SDMs is relatively novel in public health. Even though SAs are gaining momentum—especially in relation to non-communicable diseases^12^—the value of the integration of system dynamics modelling is still being scrutinised. Typically, what an SDM can offer to SAs in public health is perceived to lie broadly in one of two objectives. The SDM is developed together with stakeholders as part of GMB to, in the process, shift their thinking from individuals to systems. Alternatively, the SDM is prepared by researchers as a decision-making tool to test interventions, the results of which can inform policy, where the SDM is finetuned to the extent that it can examine the results of concretely delineated actions.^38 39^ This study contributes to the literature by suggesting that there might also be a third objective. The small and high-level SDM, using what-if scenarios reflecting interventions on different system levels, could contribute to understanding and communicating where systems thinking may get us: what if we did things differently? The use of local data can amplify this aim, providing “an idea of where we could end up”. An SDM could complement insights gained through systems maps and GMB, as it helps shift the focus from understanding the system to changing it. It seems clear that a presentation of simulation results cannot replace GMB when it comes to the cultivation of systems thinking skills, but could support telling the story in a concise and hopeful manner to third-party stakeholders. Through simulation, the complexity of all dynamics relevant to the problem can be condensed into one outcome of interest and the possible effects of intervening on higher system levels can be concretised.

Our results are reflective of a preliminary exploration, with a group of stakeholders that were already systems science enthusiasts, but do illustrate how SDMs, even if they are small and high-level, could contribute to (community-based) SAs in public health when it comes to communicating the potential of systems thinking. What we learnt is that calibration of an existing SDM with data of a different population is probably more feasible if the SDM is small and high-level, with general mechanisms (ie, social norms and individual behaviour interact), than if it has many factors and interconnections and is highly specific to a situation. This highlights both the advantages and disadvantages of a small and high-level SDM. On the one hand, because of its more general applicability, it allows for the replication of simulation results across populations and could help ‘recycle’ models across SAs, providing a starting point for others that could then be tailored to the local context. On the other hand, while such a small and high-level SDM can be used to show *why* an SA is necessary—that is, communicating the idea—it does not suffice if the goal is to infer *what* actions should exactly be taken, a limitation that was also reflected on in the workshop. These simulation results can be used to illustrate that unhealthy social norms may diminish the effectiveness of individual-level interventions. The feedback effects between environment-level social norms and individual-level eating and physical activity behaviour, and the what-if scenarios, were chosen as an example: so as to reflect the interconnectedness between drivers of population health across multiple levels of society, and the possible effects of intervening on these different system levels.^31^ The SDM does not give a comprehensive overview of factors that are relevant to addressing childhood obesity (in each local context) and is not equipped to indicate which precise actions are likely to improve system behaviour. Quantification of a systems map may not be viable in every SA, where it is a longstanding discussion in the field of system dynamics whether simulation is necessary for each purpose an SA may have.^2940^,^42^ A small and high-level SDM, calibrated with local data, could provide a solution when it comes to the purpose of communicating the idea and “aha moment[s]”.

### Future research

The same research question should be explored within other SAs in public health, with stakeholders new to systems science, to assess how much of the results of this preliminary exploration are dependent on the prior knowledge of the stakeholders consulted. We also wonder to what extent the SDM would have been valuable to the stakeholders if we had not calibrated it with local data, but presented it as an illustration of intervening on different system levels—although in another population.

## Conclusions

Our preliminary exploration showed that the small and high-level SDM, using what-if scenarios reflecting interventions on different system levels, could contribute to the understanding and communication of (community-based) SAs.

## supplementary material

10.1136/bmjopen-2024-087195 online supplemental file 1

## Data Availability

Data are available upon reasonable request.
