# Supplementary material for: Exploring the value Australian community leaders see in a system dynamics model calibrated with local data: social norms and childhood obesity
Source: BMJ Open. 2025 Feb 19;15(2):e087195. doi: 10.1136/bmjopen-2024-087195 (PMC11840901; doi:10.1136/bmjopen-2024-087195)
Supplement: online supplemental file 1 [file bmjopen-15-2-s001.docx]

**Supplementary Material**

**Exploring the value Australian community leaders see in a system dynamics model calibrated with local data: Social norms and childhood obesity**

Loes Crielaard, PhD^1^, Andrew D Brown, MSW^2^, Mary Nicolaou, PhD^1^, Joshua Hayward, PhD^2^, Prof. Karien Stronks, PhD^1^, Prof. Steven Allender, PhD^2^

^1^Amsterdam University Medical Centres, Department of Public and Occupational Health, Amsterdam, The Netherlands
^2^Faculty of Health, Global Centre for Preventive Health and Nutrition (GLOBE), Institute for Health Transformation, Deakin University, Geelong, Victoria, Australia

**Correspondence to:** Loes Crielaard, PhD, Amsterdam University Medical Centres, Department of Public and Occupational Health, Amsterdam, The Netherlands, l.crielaard@amsterdamumc.nl

**Table of contents**

1. **WHOSTOPS data** 1
2. **System dynamics model** 2
   1. ***Values imported into system dynamics model estimated from WHOSTOPS data*** 2
   2. ***Changes to optimisation procedure compared to existing system dynamics model*** 3
   3. ***Parameter values* Intent_EB_ *and* Intent_PAB_** 4
   4. ***Simulation results*** 5
3. **References** 5

1. **WHOSTOPS data**

Weight and height were measured privately by health professionals while the children wore light clothing and no shoes.^1^ Two measurements were taken for both weight and height (to the nearest 0.1 kg and 0.1 cm, respectively) – a third measurement was taken if there was a discrepancy between the two initial measurements.^1^ Average weight and height were estimated for each child across these measurements.^1^ Time spent in moderate-to-vigorous physical activity over the previous seven days was self-reported by the children and used to determine adherence (≥ 60 minutes per day of moderate-to-vigorous physical activity) to the physical activity component of Australia’s 24-hour movement guidelines.^1^ Children self-reported weight perception by responding to the question ‘How would you describe your weight?’, where the answers that could be selected were ‘Very underweight’, ‘Slightly underweight’, ‘About the right weight’, ‘Slightly overweight’, and ‘Very overweight’.^2^

2. **System dynamics model**

2.1. ***Values imported into system dynamics model estimated from WHOSTOPS data***

Physical activity level at baseline was determined based on gender, age, and adherence to the physical activity component of Australia’s 24-hour movement guidelines five out of seven days per week using the Human Energy Requirements report^3^ (in report: page 29, Table 4.5 for boys; page 30, Table 4.6 for girls). Specifically, weighted averages based on distribution of gender and age across the sample were calculated to correspond to light physical activity level (1.46), used as physical activity level at baseline if a child did not adhere to the guideline, and to moderate physical activity level (1.7), used if a child did adhere to the guideline.

The value for healthy BMI (16.82 kg/m^2^) was calculated as a weighted average based on distribution of gender and age across the sample using BMI-for-age growth reference tables by the World Health Organization^4^ and was identical for all children (values taken from 1^st^ month of an age group). Total daily energy intake was assumed to be equal to total daily energy expenditure at baseline, where total daily energy expenditure was determined based on basal metabolic rate, which can be calculated when age and weight are known, and physical activity level using equation (5) (Table 1 in the main paper).^5^

Socio-cultural ideal BMI was estimated from weight perception, which was self-reported based on the question ‘How would you describe your weight?’ with possible answers ‘Very underweight’, ‘Slightly underweight’, ‘About the right weight’, ‘Slightly overweight’, and ‘Very overweight’.^2^ First, stratified by community, each answer was mapped to a corresponding average measured BMI – calculated over the subsample of children with that answer. Second, each individual’s socio-cultural ideal BMI was estimated by taking their measured BMI and summing it with the difference between the average measured BMI of their weight perception subsample and the average measured BMI of the ‘About the right weight’ subsample. For example, if an individual self-reports as ‘Very underweight’ and has a measured BMI of 20 kg/m^2^ and the difference between the ‘Very underweight’ subsample and the ‘About the right weight’ subsample in their community is -0.94 kg/m^2^, it is deduced that their socio-cultural ideal BMI corresponds to their measured BMI plus this difference: 19.06 kg/m^2^. This assumes that all children would prefer ‘About the right weight’ and relies on weight perception not necessarily correlating with measured BMI. The procedure results in a socio-cultural ideal BMI value for each individual that is relative to their community.

2.2. ***Changes to optimisation procedure compared to existing system dynamics model***

To calibrate the system dynamics model we made a few minor changes as compared to the original method.^5^ The first change is that the alpha parameter (as elaborated upon in Supplementary Material accompanying the previously published system dynamics model^5^) is now defined as the percent weight change per time step towards the population average. Previously it was defined as the number of time steps needed to reach the population average when assuming a linear progression (constant weight change per time step). The new definition is more accurate since in a system dynamics model with linear interactions the progressions are actually exponential.

The second change is that the alpha parameter is now computed in such a way that the *average* weight change equals 1 kg per month, towards the population average and in the initial time step. In the original method the alpha parameter was computed such that the *maximum* weight change equals 2 kgs per month. The problem with the original approach would be that the variation in weight within the current population is very large, since it consists of children that vary in age and gender – making the maximum prone to outliers. We opted for the average weight change to equal 1 kg per month because 2 kgs per month is not reflective of healthy weight loss for children. The choice of 1 kg per month is in a sense arbitrary but also irrelevant, as the parameter values for *Intent_EB_* and *Intent_PAB_* for each community depend linearly on this parameter. That is, if we would have chosen 0.5 kg of average weight change per month instead of 1 kg, then the parameter values for both *Intent_EB_* and *Intent_PAB_* (see Table S.1 below) would also become 50% of their original values. In other words, visually, this change would result in the same simulated trajectories as in Figure 2 in the main paper except that they would be stretched in the x-axis direction (by a factor of 2). In summary, such parameter changes (as long as they remain in plausible ranges) do not alter the simulation results in terms of the ordering of the three different scenario lines and do not significantly alter the relative differences between the lines.

The third change is that we added a penalty to the optimisation procedure that ensured that the parameter value for *Intent_EB_* could not become smaller than -350 (kcal/day)/(kg/m^2^). The problem that the penalty solves is that for the optimisation procedure it would be equally ‘good’ to let individuals overshoot their movement towards the population average. That is, if an individual is meant to change 10% of their difference in weight with the population average, then that same lower distance to the average would equally be achieved by changing 190%. Although this is not biologically plausible, the optimisation procedure currently has no sense of biological plausibility implemented and could sometimes choose such an oscillatory solution. The added penalty prevents such solutions. This penalty was not required for the Amsterdam-based adult cohort as the variation in weight was not as large as within the current population.

The fourth and final change is that we now determine the 95% confidence interval non-parametrically by bootstrapping 10,000 medians per time step for each curve. The reason is that the original method – using a direct formula for the confidence interval of medians, which assumes that the data are roughly binomially distributed – did not provide accurate intervals, when compared to the outcomes of the bootstrap method (which makes no such assumption). Another direct formula to determine the 95% confidence interval, which assumes that the data are normally distributed, also led to significant deviations to the bootstrap method. Therefore we decided to fall back to the (more computationally expensive) bootstrap method.

2.3. ***Parameter values* Intent_EB_ *and* Intent_PAB_**

Table S.1 shows the optimal parameter values for *Intent_EB_* and *Intent_PAB_* for each community as estimated according to the optimisation procedure described in the Supplementary Material accompanying the previously published system dynamics model.^5^ These parameter values are plugged into the equations described in Table 1 in the main paper to generate the simulation results. *Intent_EB_* and *Intent_PAB_* refer to the change made in eating behaviour and physical activity level, respectively, and are expressed as a change in total daily energy intake and physical activity level per day per unit of BMI that their BMI differs from their individual ideal BMI.^5^

***Table S.1. Optimisation results for each community.***

|  | *Intent_EB_* in (kcal/day)/(kg/m^2^) | *Intent_PAB_* in 1/(kg/m^2^) |
| --- | --- | --- |
| Community 1 | -80.95 | 0.021 |
| Community 2 | -68.59 | 0.018 |
| Community 3 | -74.03 | 0.02 |
| Community 4 | -79.01 | 0.02 |
| Community 5 | -94.27 | 0.025 |
| Community 6 | -90.54 | 0.024 |
| Pooled | -82.68 | 0.021 |

2.4. ***Simulation results***

Table S.2 shows the community-level median BMI at t=0, as directly extracted from the WHOSTOPS data, and the estimated community-level median BMI at t=36 (end of the simulation) for the what-if scenarios corresponding to implementing no intervention, implementing an individual-level intervention, and implementing the same individual-level intervention combined with an environment-level intervention.

***Table S.2. Simulation results for each community.***

|  | | Community 1 | Community 2 | Community 3 | Community 4 | Community 5 | Community 6 | Pooled |
| --- | --- | --- | --- | --- | --- | --- | --- | --- |
| Community-level median BMI (kg/m^2^) at t=0 | | 18.77 | 18.48 | 18.14 | 18.43 | 18.10 | 18.21 | 18.21 |
| No intervention | Community-level median BMI (kg/m^2^) at t=36 | 18.77 | 18.18 | 18.14 | 18.34 | 18.06 | 18.09 | 18.20 |
| Individual-level intervention | Community-level median BMI (kg/m^2^) at t=36 | 17.95 | 17.77 | 17.56 | 17.70 | 17.58 | 17.59 | 17.68 |
| Individual-level intervention combined with environment-level intervention | Community-level median BMI (kg/m^2^) at t=36 | 17.50 | 17.48 | 17.19 | 17.40 | 17.24 | 17.23 | 17.31 |

3. **References**

1. Allender S, Orellana L, Crooks N, et al. Four‐Year Behavioral, Health‐Related Quality of Life, and BMI Outcomes from a Cluster Randomized Whole of Systems Trial of Prevention Strategies for Childhood Obesity. *Obesity* 2021;29(6):1022-35. doi: 10.1002/oby.23130

2. Hayward J, Millar L, Petersen S, et al. When ignorance is bliss: weight perception, body mass index and quality of life in adolescents. *International Journal of Obesity* 2014;38(10):1328-34. doi: 10.1038/ijo.2014.78

3. FAO, WHO, UNU. Human Energy Requirements. Rome, 2001:1-105.

4. World Health Organization. Growth reference data for 5-19 years. BMI-for-age (5-19 years). Tables. Percentiles, 2007.

5. Crielaard L, Dutta P, Quax R, et al. Social norms and obesity prevalence: From cohort to system dynamics models. *Obesity Reviews* 2020;21(9):1-17. doi: 10.1111/obr.13044
