# Supplementary material for: Exploring the value Australian community leaders see in a system dynamics model calibrated with local data: social norms and childhood obesity
Source: BMJ Open. 2025 Feb 19;15(2):e087195. doi: 10.1136/bmjopen-2024-087195 (PMC11840901; doi:10.1136/bmjopen-2024-087195)
Supplement: Supplementary file 2 [file bmjopen-2024-087195.reviewer_comments.pdf]

# PEER REVIEW HISTORY

BMJ Open publishes all reviews undertaken for accepted manuscripts. Reviewers are asked to complete a checklist review form and are provided with free text boxes to elaborate on their assessment. These free text comments are reproduced below.

## ARTICLE DETAILS

### Title (Provisional)

Exploring the value Australian community leaders see in a system dynamics model calibrated with local data: Social norms and childhood obesity

### Authors

Crielaard, Loes; Brown, Andrew; Nicolaou, Mary; Hayward, Joshua; Stronks, Karien; Allender, Steven

---

## VERSION 1 - REVIEW

---

| | |
|---|---|
| **Reviewer** | **1** |
| **Name** | **Nobles, James** |
| **Affiliation** | **Leeds Beckett University** |
| **Date** | **31-May-2024** |
| **COI** | **n/a** |

---

This is a very topical paper which provides information on how to advance work within the field of systems approaches, particularly in the context of obesity prevention. As the authors rightly state, much of the work to date is limited given that it relies heavily on qualitative systems maps. This paper therefore seeks to advance our thinking, by taking a causal loop diagram, drawing on local data, to create a systems dynamics model. They tested the application of the SDM with a range of community leaders.

I cannot comment on the processes related to the SDM development as this is outside of my expertise, however I can comment on the paper at a high level and based on the qualitative insight work with the community leaders.

I feel there are several issues which need to be addressed before being considered for publication.

1. The introduction and discussion tell quite a contrasting story. In the introduction, you highlight some of the limitations of GMB (e.g. resource and expertise intensive) and use this as a rationale to suggest that SDM may be a useful alternative (i.e. less reliant on external experts, people can take the model and run simulations for themselves). In the discussion, you state that the SDM is still reliant on academic expertise and is resource intensive, and

that you do not know whether it can be used to simulate scenarios without academic expertise. So this doesnt quite line up.

I think the issue lies in the introduction and the framing of the study. Perhaps focus less on the limitations of GMB, and positioning SDM as an alternative, and instead state how SDM can build on what we know from GMB. Highlight the strengths of both, and say how they complement one another (you actually say this on line 399 in the results). I think the purpose of SDM is quite different to GMB, and that it shouldnt therefore be positioned as an either / or. I would also remove the reference in the introduction to SDM being a useful tool that doesnt require academic expertise (lines 114/115). The paragraph from lines 90 - 107 is the one most in need of careful consideration. There are other ways, which would be less resource intensive than GMB and SDM, to communicate the complexity or systemic nature of a problem.

2. Bias within the workshop sample. You have addressed this in the limitations, but I am not sure if you have done this sufficiently. You are starting with a biased sample of 100 people who are highly engaged in local systems approaches, and then recruit arguably the 11 most engaged / interested individuals in the workshop (they reference Donella Meadows, which is a testament to the approach, but highlights that this is not your typical sample). I'm not convinced therefore that we get a balanced perspective from their feedback about the utility of SDM, especially when we consider this against the rationale for SDM in the introduction. Perhaps seeing this more as a pilot, and being more careful in your conclusions, would help balance the paper out in light of the sampling bias. For example, I am not convinced that we could take an SDM to a community group who have no experience of systems approaches, and expect this to give them a systems perspective. I appreciate the subjectiveness of my comment too, but the arguments put forward in the discussion and the conclusions drawn feel overstated in light of the sampling bias.

3. What are the limitations of the SDM and were these picked up on by the stakeholder group? This SDM only focuses on a very small part of the puzzle, "norms", and so how does the SDM account for this? My main concern here is how does the SDM align with the need to change multiple aspects of a system simultaneously? Does it allow people to truly think about systems dynamics or does it push them more towards thinking about single interventions which may seek to change “norms”? This is perhaps more of a conceptual concern rather than one based on your methodology / results, but could be helpful to discuss / highlight in the discussion / future recommendations. What would the next step be for SDM? Is it feasible to build up a comprehensive SDM aligned to a complete CLD for what is driving obesity at the local level, and to model how several actions may create change across the system? I don’t think this type of question is within the remit of the study per se, but more of a consideration for future work.

Minor comments

- Typos on lines 91 and 116, with the use of e.g. mid-sentence - remove the e.g..

| | |
|---|---|
| **Reviewer** | **2** |
| **Name** | **Barbrook-Johnson, Peter** |
| **Affiliation** | **University of Oxford** |
| **Date** | **21-Jun-2024** |
| **COI** | **None** |

Summary: this paper explores the use of a SD model as a communication tool and as a tool to aid systems thinking among a group of stakeholders. It presents the model and a workshop in which it was used, with feedback from participants.

Main comments: this is a very well-written and executed paper, it is clear and concise. The aim is interesting and useful, but I am not sure it is that novel – researchers have argued for some time, for the use of many different types of models in this way (including myself), and shown examples with user feedback. This is not a fatal issue I think, but I do have a few main concerns, they are:

1. The methodology is not sufficient to answer the question - it is not enough to run one quite small workshop and gather feedback from participants, and not much else, even if you do it very well. This makes the contribution of the paper quite thin in my opinion.

2. The paper fails to develop a wider thesis or advice on the use of SD models in this mode – there is not enough discussion about how SD models should be adapted to be used in this way, or what unique qualities SD models have for this role. This would have helped neutralise my first concern (it is how I have dealt with this issue myself). More conceptual discussion, and practical advice and guidance would help, throughout. You could make space for this by not presenting the actual model results, which are not central to your purpose, or at least shorten them, or put them in an appendix.

3. The premise, that a SD model is quicker to use than GMB is entirely reliant on there being a usable SD model already in existence for a topic, this will not be the case most of the time. This is not discussed, and somewhat undermines the contribution. In the intro, the difference in purpose and outputs of these two approaches is also not explained enough, I think you underplay the difference. Only later in the paper is the discussion of the purpose of using a SD model like this nuanced enough on this point – see my more specific comment below.

4. There is little discussion of the large participatory modelling and GMB literatures, where these issues are often discussed. Given my first and second points above, I think some summary of the wider literature here, not just those immediately connected to public health, or this policy area, are needed. I would start with the 'Companion Modelling', GMB

in other policy areas, and participatory modelling literatures. I know these best in the environmental domain, where there is lots of useful stuff others have done in similar veins.

I hope the authors agree these are constructive comments, I believe they will make the paper much stronger and useful for other readers.

More specific comments:

• Abstract – in the results section of the abstract the emphasis shifts a bit, towards commenting on the model's findings. I would suggest sticking just to the role of the model as a communication tool, and add another finding or two on this front.

• Introduction – well written and clear. Literature cited is quite narrow - missing literature on use of CLD and SD as communication tools. Missing wider lit on the use of models as communication and participatory discussion tools. No discussion of the potential difference in purpose between GMB and presenting an SD.

• Methods - very clear and thorough

• Results - I don't know why you present the simulation results, they are not that relevant to your question. I would reduce this a lot and then make space for more of the discussion and literature I suggest above.

• Discussion - Page 24, lines 468 onwards – here the discussion is much better. I think some of this framing should come up to the introduction and abstract.

---

## VERSION 1 - AUTHOR RESPONSE

| Reviewer 1: Dr. James Nobles, Leeds Beckett University, University of Bristol Medical School | |
|---|---|
| 1. This is a very topical paper which provides information on how to advance work within the field of systems approaches, particularly in the context of obesity prevention. As the authors rightly state, much of the work to date is limited given that it relies heavily on qualitative systems maps. This paper therefore seeks to advance our thinking, by taking a causal loop diagram, drawing on local data, to create a systems dynamics model. They tested the application of the SDM with a range of community leaders.<br><br>I cannot comment on the processes related to the SDM development as this is outside of my expertise, however I can comment on the paper at a high level and based on the qualitative insight work with the community leaders. | We appreciate Dr. Nobles' efforts in reviewing the manuscript. His comments have been very helpful to us in strengthening the manuscript, as we present in detail below. |

| | | |
|---|---|---|
| | I feel there are several issues which need to be addressed before being considered for publication. | |
| 2. | The introduction and discussion tell quite a contrasting story. In the introduction, you highlight some of the limitations of GMB (e.g. resource and expertise intensive) and use this as a rationale to suggest that SDM may be a useful alternative (i.e. less reliant on external experts, people can take the model and run simulations for themselves). In the discussion, you state that the SDM is still reliant on academic expertise and is resource intensive, and that you do not know whether it can be used to simulate scenarios without academic expertise. So this doesnt quite line up.<br><br>I think the issue lies in the introduction and the framing of the study. Perhaps focus less on the limitations of GMB, and positioning SDM as an alternative, and instead state how SDM can build on what we know from GMB. Highlight the strengths of both, and say how they complement one another (you actually say this on line 399 in the results). I think the purpose of SDM is quite different to GMB, and that it shouldnt therefore be positioned as an either / or. I would also remove the reference in the introduction to SDM being a useful tool that doesnt require academic expertise (lines 114/115). The paragraph from lines 90 - 107 is the one most in need of careful consideration. There are other ways, which would be less resource intensive than GMB and SDM, to communicate the complexity or systemic nature of a problem. | We agree with Dr. Nobles that the framing of the study needed improvement, and we have struggled with how to present it in the best way possible in previous versions. His comments have really helped everything fall into place, as we hope he agrees. We have adopted the suggestions in rewriting the introduction to more closely align with the discussion, focusing on the strengths of both GMB and SDM and their complementary qualities (page 4-9). |
| 3. | Bias within the workshop sample. You have addressed this in the limitations, but I am not sure if you have done this sufficiently. You are starting with a biased sample of 100 people who are highly engaged in local systems approaches, and then recruit arguably the 11 most engaged / interested individuals in the workshop (they reference Donella Meadows, which is a testament to the approach, but highlights that this is not your typical sample). I'm not convinced therefore that we get a balanced perspective from their feedback about the utility of SDM, especially when we consider this against the rationale for SDM in the introduction. Perhaps seeing this more as a pilot, and being more careful in your conclusions, would help balance the paper out in light of the sampling bias. For example, I am not convinced that we could take an SDM to a community | On top of the adjustments that we made in light of comment 4, reframing the study and nuancing the findings, we have emphasised in the abstract, the introduction and the discussion that this was a “preliminary exploration” and have highlighted the future research that is required given the sampling bias of this study at the end of the manuscript (page 33, line 658). |

| | |
|---|---|
| group who have no experience of systems approaches, and expect this to give them a systems perspective. I appreciate the subjectiveness of my comment too, but the arguments put forward in the discussion and the conclusions drawn feel overstated in light of the sampling bias. | |
| 4. What are the limitations of the SDM and were these picked up on by the stakeholder group? This SDM only focuses on a very small part of the puzzle, "norms", and so how does the SDM account for this? My main concern here is how does the SDM align with the need to change multiple aspects of a system simultaneously? Does it allow people to truly think about systems dynamics or does it push them more towards thinking about single interventions which may seek to change "norms"? This is perhaps more of a conceptual concern rather than one based on your methodology / results, but could be helpful to discuss / highlight in the discussion / future recommendations. What would the next step be for SDM? Is it feasible to build up a comprehensive SDM aligned to a complete CLD for what is driving obesity at the local level, and to model how several actions may create change across the system? I don't think this type of question is within the remit of the study per se, but more of a consideration for future work. | We have clarified in the improved framing of the study that the SDM's focus on social norms was meant as an illustrative example of feedback effects between the social environment and individual behaviour, and that this SDM did not have the purpose of supporting decision-making – as it does not offer options of concretely delineated actions: it is just used to communicate the idea of intervening on different system levels. We have alluded to this purpose throughout the manuscript now, and reflect on this point in the discussion (page 30-32). |
| 5. Typos on lines 91 and 116, with the use of e.g. mid-sentence - remove the e.g.. | Thank you! |
| **Reviewer 2: Dr. Peter Barbrook-Johnson, University of Oxford** | |
| 6. Summary: this paper explores the use of a SD model as a communication tool and as a tool to aid systems thinking among a group of stakeholders. It presents the model and a workshop in which it was used, with feedback from participants.<br><br>Main comments: this is a very well-written and executed paper, it is clear and concise. The aim is interesting and useful, but I am not sure it is that novel – researchers have argued for some time, for the use of many different types of models in this way (including myself), and shown examples with user feedback. This is not a fatal issue I think, but I do have a few main concerns, they are: | We thank Dr. Barbrook-Johnson for his efforts in reviewing the manuscript and appreciate his positive feedback. The comments were of great help in improving and clarifying the manuscript. Many of the comments made by Dr. Barbrook-Johnson overlapped with the comments of Dr. Nobles, so below we will often refer to earlier comments to show how we addressed them to resolve the concerns of both Dr. Barbrook-Johnson and Dr. Nobles. |

| | | |
|---|---|---|
| 7. | The methodology is not sufficient to answer the question - it is not enough to run one quite small workshop and gather feedback from participants, and not much else, even if you do it very well. This makes the contribution of the paper quite thin in my opinion. | We agree that the framing of our study needed to be adjusted somewhat and Dr. Barbrook-Johnson's comments have been of great help in seeing how to best present what we did – as we hope he agrees. We refer to comment 4 and comment 5 above for how we addressed this. |
| 8. | The paper fails to develop a wider thesis or advice on the use of SD models in this mode – there is not enough discussion about how SD models should be adapted to be used in this way, or what unique qualities SD models have for this role. This would have helped neutralise my first concern (it is how I have dealt with this issue myself). More conceptual discussion, and practical advice and guidance would help, throughout. You could make space for this by not presenting the actual model results, which are not central to your purpose, or at least shorten them, or put them in an appendix. | We would again like to refer to comment 4 and comment 5, and have taken the suggestion of not presenting the actual model results as a main research outcome. We have now more elaborately reflected on the qualities of the SDM, and what it can and cannot do, in the discussion (page 30-32). |
| 9. | The premise, that a SD model is quicker to use than GMB is entirely reliant on there being a usable SD model already in existence for a topic, this will not be the case most of the time. This is not discussed, and somewhat undermines the contribution. In the intro, the difference in purpose and outputs of these two approaches is also not explained enough, I think you underplay the difference. Only later in the paper is the discussion of the purpose of using a SD model like this nuanced enough on this point – see my more specific comment below. | We agree that reframing of the study was required: we have adopted the suggestions in rewriting the introduction to more closely align with the discussion, focusing on the strengths of both GMB and SDM and their complementary qualities (page 4-9) (see also comment 4). |
| 10. | There is little discussion of the large participatory modelling and GMB literatures, where these issues are often discussed. Given my first and second points above, I think some summary of the wider literature here, not just those immediately connected to public health, or this policy area, are needed. I would start with the 'Companion Modelling', GMB in other policy areas, and participatory modelling literatures. I know these best in the environmental domain, where there is lots of useful stuff others have done in similar veins. | We have now taken care to refer to the wider literature on GMB and simulation, which we knew best in the system dynamics realm. We summarise contributions by Sterman, Hovmand, Meadows and Vennix and have incorporated reviews on GMB effectiveness by Scott and Rouwette. While not engaging with the debate ourselves on this occasion, we |

| | |
|---|---|
| I hope the authors agree these are constructive comments, I believe they will make the paper much stronger and useful for other readers. | have also referred to relevant literature on qualitative and quantitative system dynamics, which each may serve different purposes, in the discussion (page 32). |
| 11. Abstract – in the results section of the abstract the emphasis shifts a bit, towards commenting on the model's findings. I would suggest sticking just to the role of the model as a communication tool, and add another finding or two on this front. | We have taken this suggestion (in line with comment 10). |
| 12. Introduction – well written and clear. Literature cited is quite narrow - missing literature on use of CLD and SD as communication tools. Missing wider lit on the use of models as communication and participatory discussion tools. No discussion of the potential difference in purpose between GMB and presenting an SD. | We have addressed these issues in comment 4 and comment 12. |
| 13. Methods - very clear and thorough | Thank you, we have left the methods largely unchanged. |
| 14. Results - I don't know why you present the simulation results, they are not that relevant to your question. I would reduce this a lot and then make space for more of the discussion and literature I suggest above. | We have taken this suggestion (in line with comment 10 and comment 13). |
| 15. Discussion - Page 24, lines 468 onwards – here the discussion is much better. I think some of this framing should come up to the introduction and abstract. | We have worked on this, as presented in comment 4. |
